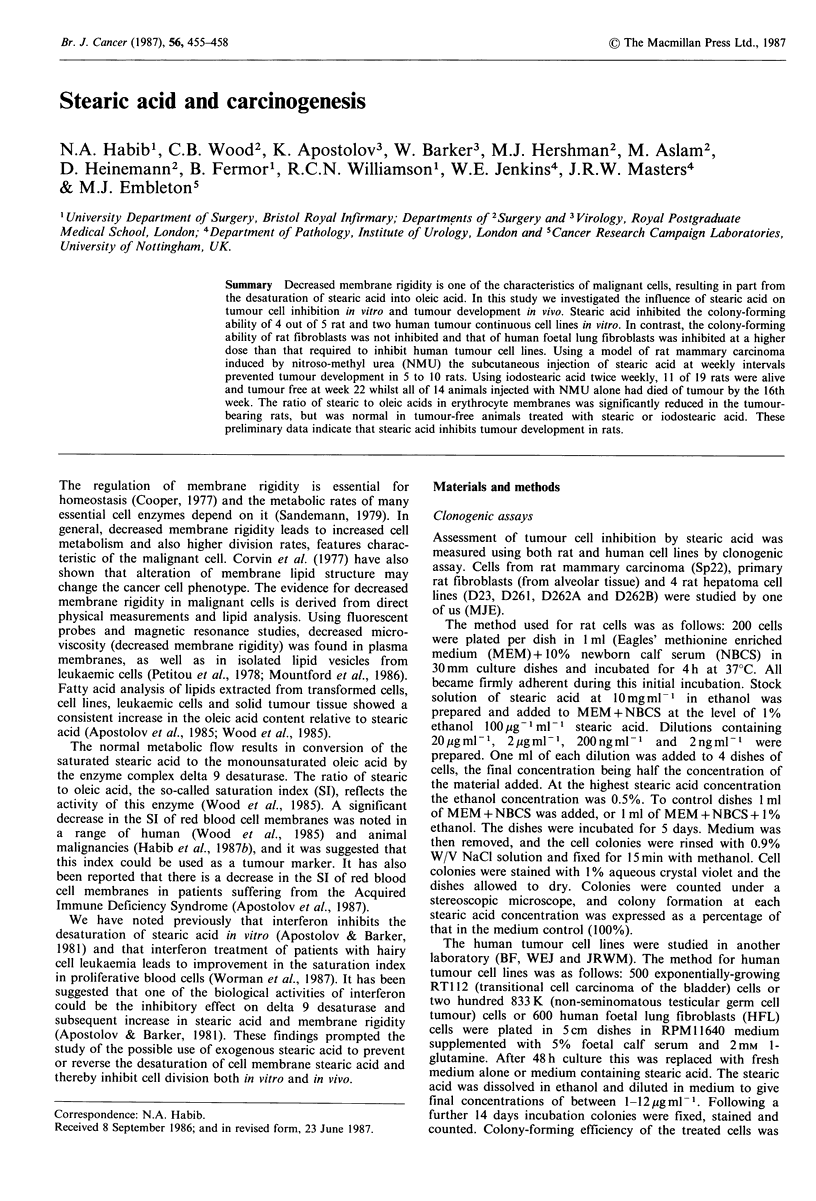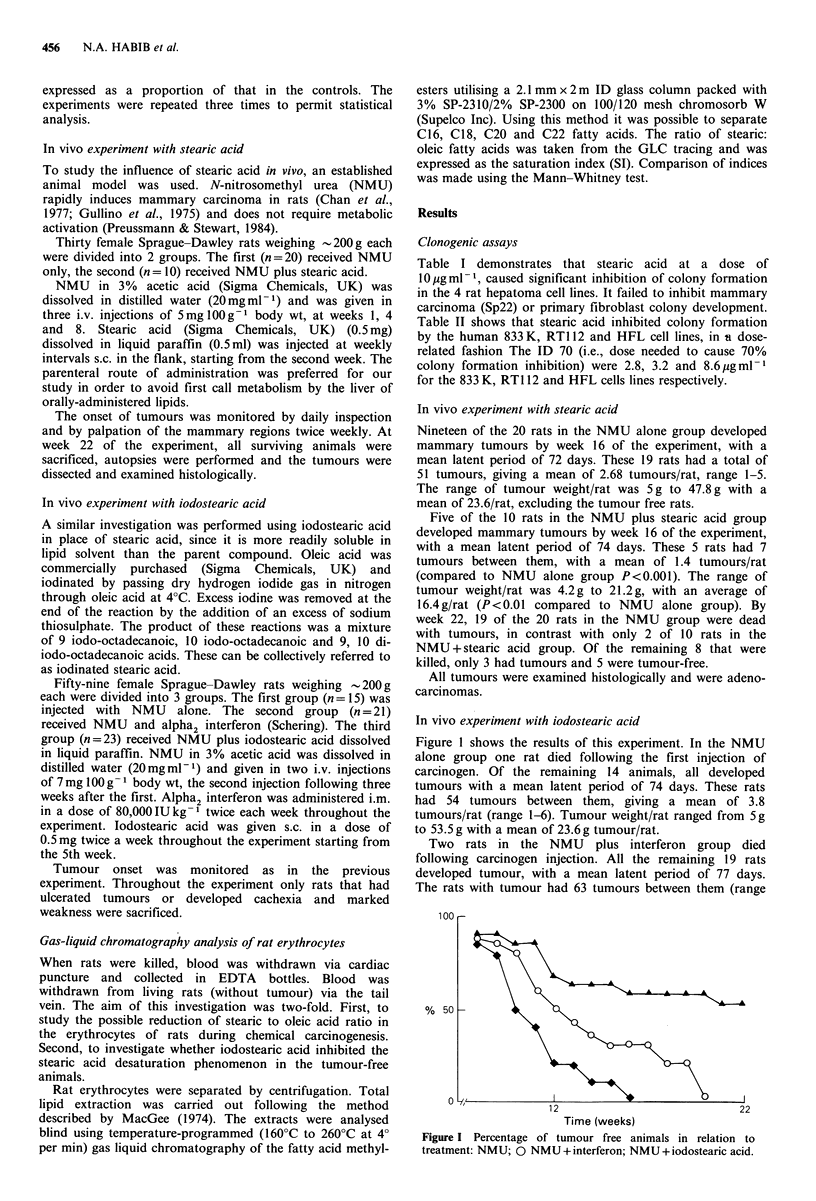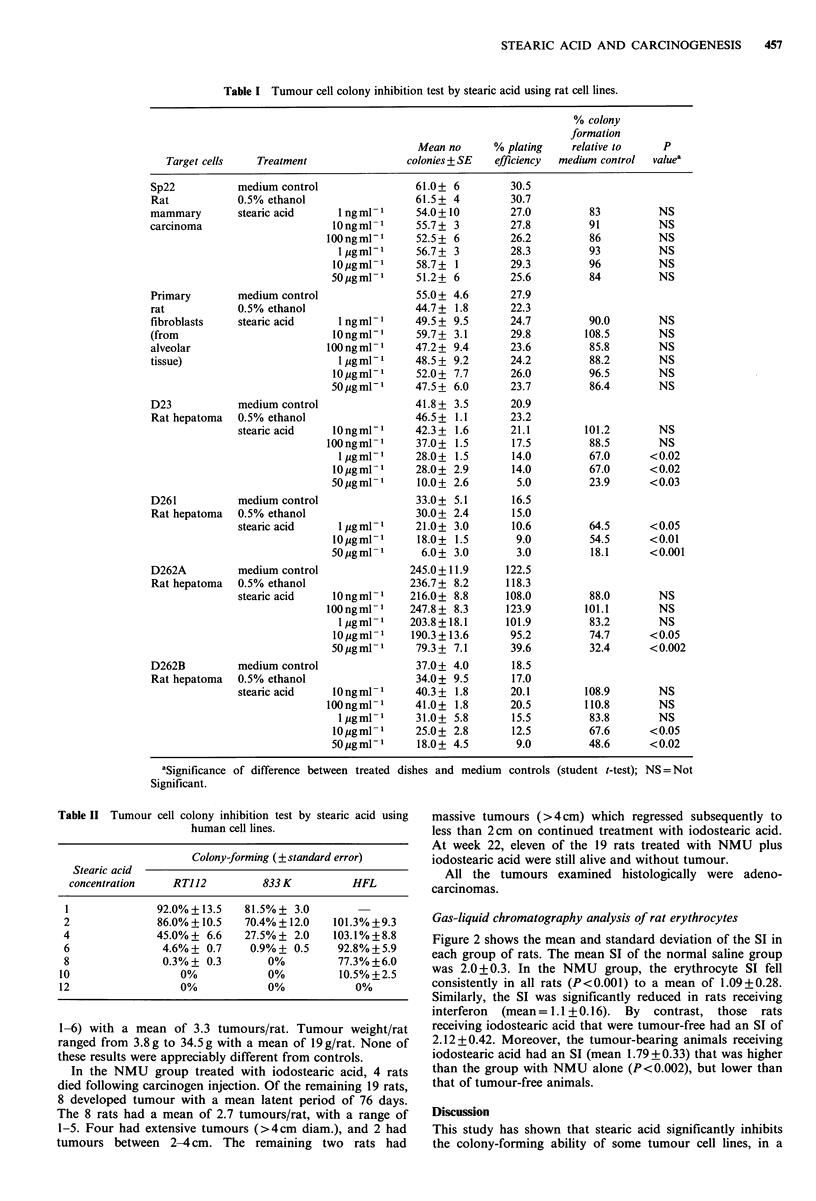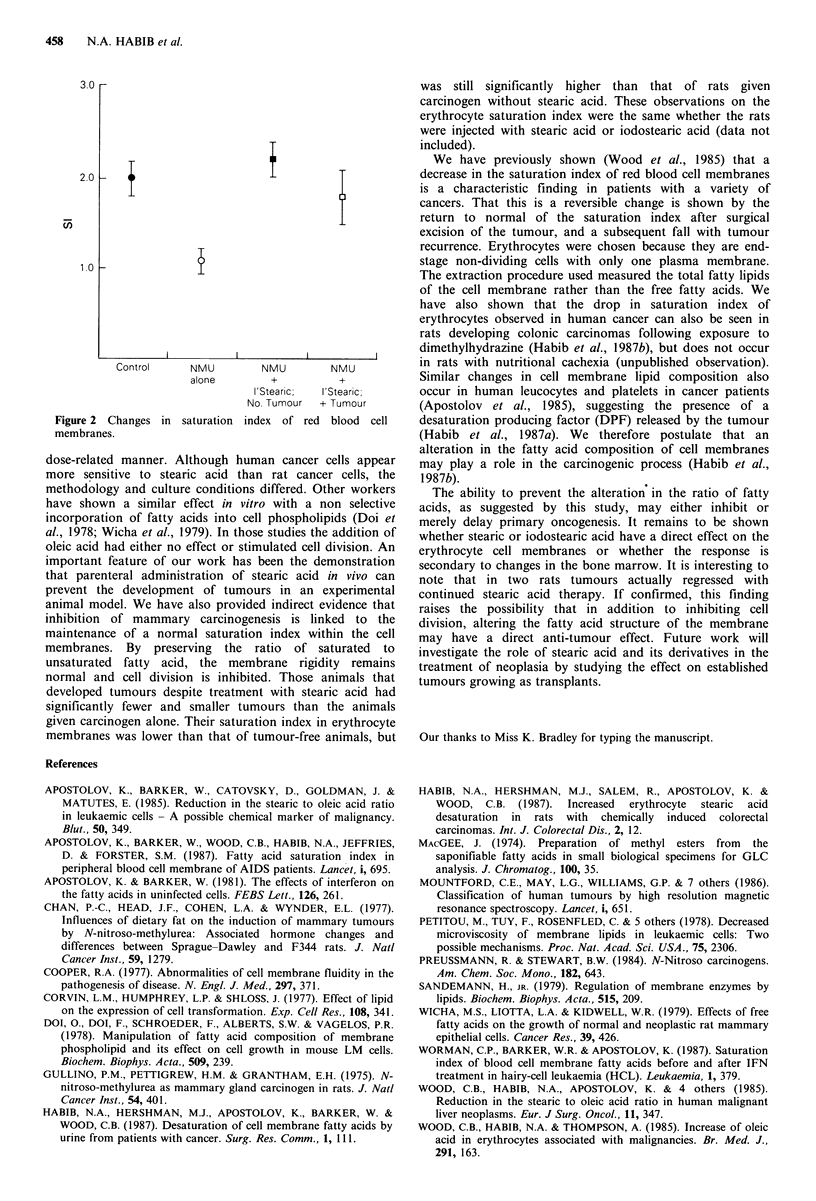# Stearic acid and carcinogenesis.

**DOI:** 10.1038/bjc.1987.223

**Published:** 1987-10

**Authors:** N. A. Habib, C. B. Wood, K. Apostolov, W. Barker, M. J. Hershman, M. Aslam, D. Heinemann, B. Fermor, R. C. Williamson, W. E. Jenkins

**Affiliations:** University Department of Surgery, Bristol Royal Infirmary, UK.

## Abstract

Decreased membrane rigidity is one of the characteristics of malignant cells, resulting in part from the desaturation of stearic acid into oleic acid. In this study we investigated the influence of stearic acid on tumour cell inhibition in vitro and tumour development in vivo. Stearic acid inhibited the colony-forming ability of 4 out of 5 rat and two human tumour continuous cell lines in vitro. In contrast, the colony-forming ability of rat fibroblasts was not inhibited and that of human foetal lung fibroblasts was inhibited at a higher dose than that required to inhibit human tumour cell lines. Using a model of rat mammary carcinoma induced by nitroso-methyl urea (NMU) the subcutaneous injection of stearic acid at weekly intervals prevented tumour development in 5 to 10 rats. Using iodostearic acid twice weekly, 11 of 19 rats were alive and tumour free at week 22 whilst all of 14 animals injected with NMU alone had died of tumour by the 16th week. The ratio of stearic to oleic acids in erythrocyte membranes was significantly reduced in the tumour-bearing rats, but was normal in tumour-free animals treated with stearic or iodostearic acid. These preliminary data indicate that stearic acid inhibits tumour development in rats.


Br. J. Cancer (1987), 56, 455–458 © The Macmillan Press Ltd., 1987

### Stearic acid and carcinogenesis

N.A. Habib[1], C.B. Wood[2], K. Apostolov[3], W. Barker[3], M.J. Hershman[2], M. Aslam[2], D. Heinemann[2], B. Fermor[1], R.C.N. Williamson[1], W.E. Jenkins[4], J.R.W. Masters[4] & M.J. Embleton[5]

[1]*University Department of Surgery, Bristol Royal Infirmary; Departments of* [2]*Surgery and* [3]*Virology, Royal Postgraduate Medical School, London;* [4]*Department of Pathology, Institute of Urology, London and* [5]*Cancer Research Campaign Laboratories, University of Nottingham, UK.*

**Summary** Decreased membrane rigidity is one of the characteristics of malignant cells, resulting in part from the desaturation of stearic acid into oleic acid. In this study we investigated the influence of stearic acid on tumour cell inhibition *in vitro* and tumour development *in vivo*. Stearic acid inhibited the colony-forming ability of 4 out of 5 rat and two human tumour continuous cell lines *in vitro*. In contrast, the colony-forming ability of rat fibroblasts was not inhibited and that of human foetal lung fibroblasts was inhibited at a higher dose than that required to inhibit human tumour cell lines. Using a model of rat mammary carcinoma induced by nitroso-methyl urea (NMU) the subcutaneous injection of stearic acid at weekly intervals prevented tumour development in 5 to 10 rats. Using iodostearic acid twice weekly, 11 of 19 rats were alive and tumour free at week 22 whilst all of 14 animals injected with NMU alone had died of tumour by the 16th week. The ratio of stearic to oleic acids in erythrocyte membranes was significantly reduced in the tumour-bearing rats, but was normal in tumour-free animals treated with stearic or iodostearic acid. These preliminary data indicate that stearic acid inhibits tumour development in rats.

The regulation of membrane rigidity is essential for homeostasis (Cooper, 1977) and the metabolic rates of many essential cell enzymes depend on it (Sandemann, 1979). In general, decreased membrane rigidity leads to increased cell metabolism and also higher division rates, features characteristic of the malignant cell. Corvin *et al.* (1977) have also shown that alteration of membrane lipid structure may change the cancer cell phenotype. The evidence for decreased membrane rigidity in malignant cells is derived from direct physical measurements and lipid analysis. Using fluorescent probes and magnetic resonance studies, decreased microviscosity (decreased membrane rigidity) was found in plasma membranes, as well as in isolated lipid vesicles from leukaemic cells (Petitou *et al.*, 1978; Mountford *et al.*, 1986). Fatty acid analysis of lipids extracted from transformed cells, cell lines, leukaemic cells and solid tumour tissue showed a consistent increase in the oleic acid content relative to stearic acid (Apostolov *et al.*, 1985; Wood *et al.*, 1985).

The normal metabolic flow results in conversion of the saturated stearic acid to the monounsaturated oleic acid by the enzyme complex delta 9 desaturase. The ratio of stearic to oleic acid, the so-called saturation index (SI), reflects the activity of this enzyme (Wood *et al.*, 1985). A significant decrease in the SI of red blood cell membranes was noted in a range of human (Wood *et al.*, 1985) and animal malignancies (Habib *et al.*, 1987*b*), and it was suggested that this index could be used as a tumour marker. It has also been reported that there is a decrease in the SI of red blood cell membranes in patients suffering from the Acquired Immune Deficiency Syndrome (Apostolov *et al.*, 1987).

We have noted previously that interferon inhibits the desaturation of stearic acid *in vitro* (Apostolov & Barker, 1981) and that interferon treatment of patients with hairy cell leukaemia leads to improvement in the saturation index in proliferative blood cells (Worman *et al.*, 1987). It has been suggested that one of the biological activities of interferon could be the inhibitory effect on delta 9 desaturase and subsequent increase in stearic acid and membrane rigidity (Apostolov & Barker, 1981). These findings prompted the study of the possible use of exogenous stearic acid to prevent or reverse the desaturation of cell membrane stearic acid and thereby inhibit cell division both *in vitro* and *in vivo*.

Correspondence: N.A. Habib.
Received 8 September 1986; and in revised form, 23 June 1987.

### Materials and methods

### *Clonogenic assays*

Assessment of tumour cell inhibition by stearic acid was measured using both rat and human cell lines by clonogenic assay. Cells from rat mammary carcinoma (Sp22), primary rat fibroblasts (from alveolar tissue) and 4 rat hepatoma cell lines (D23, D261, D262A and D262B) were studied by one of us (MJE).

The method used for rat cells was as follows: 200 cells were plated per dish in 1 ml (Eagles' methionine enriched medium (MEM) + 10% newborn calf serum (NBCS) in 30 mm culture dishes and incubated for 4 h at 37°C. All became firmly adherent during this initial incubation. Stock solution of stearic acid at $10\,mg\,ml^{-1}$ in ethanol was prepared and added to MEM + NBCS at the level of 1% ethanol $100\,\mu g^{-1}\,ml^{-1}$ stearic acid. Dilutions containing $20\,\mu g\,ml^{-1}$, $2\,\mu g\,ml^{-1}$, $200\,ng\,ml^{-1}$ and $2\,ng\,ml^{-1}$ were prepared. One ml of each dilution was added to 4 dishes of cells, the final concentration being half the concentration of the material added. At the highest stearic acid concentration the ethanol concentration was 0.5%. To control dishes 1 ml of MEM + NBCS was added, or 1 ml of MEM + NBCS + 1% ethanol. The dishes were incubated for 5 days. Medium was then removed, and the cell colonies were rinsed with 0.9% W/V NaCl solution and fixed for 15 min with methanol. Cell colonies were stained with 1% aqueous crystal violet and the dishes allowed to dry. Colonies were counted under a stereoscopic microscope, and colony formation at each stearic acid concentration was expressed as a percentage of that in the medium control (100%).

The human tumour cell lines were studied in another laboratory (BF, WEJ and JRWM). The method for human tumour cell lines was as follows: 500 exponentially-growing RT112 (transitional cell carcinoma of the bladder) cells or two hundred 833 K (non-seminomatous testicular germ cell tumour) cells or 600 human foetal lung fibroblasts (HFL) cells were plated in 5 cm dishes in RPM11640 medium supplemented with 5% foetal calf serum and 2 mM l-glutamine. After 48 h culture this was replaced with fresh medium alone or medium containing stearic acid. The stearic acid was dissolved in ethanol and diluted in medium to give final concentrations of between $1–12\,\mu g\,ml^{-1}$. Following a further 14 days incubation colonies were fixed, stained and counted. Colony-forming efficiency of the treated cells was expressed as a proportion of that in the controls. The experiments were repeated three times to permit statistical analysis.

### In vivo *experiment with stearic acid*

To study the influence of stearic acid *in vivo*, an established animal model was used. *N*-nitrosomethyl urea (NMU) rapidly induces mammary carcinoma in rats (Chan *et al.*, 1977; Gullino *et al.*, 1975) and does not require metabolic activation (Preussmann & Stewart, 1984).

Thirty female Sprague–Dawley rats weighing ~200 g each were divided into 2 groups. The first ($n=20$) received NMU only, the second ($n=10$) received NMU plus stearic acid.

NMU in 3% acetic acid (Sigma Chemicals, UK) was dissolved in distilled water ($20\,mg\,ml^{-1}$) and was given in three i.v. injections of $5\,mg\,100\,g^{-1}$ body wt, at weeks 1, 4 and 8. Stearic acid (Sigma Chemicals, UK) (0.5 mg) dissolved in liquid paraffin (0.5 ml) was injected at weekly intervals s.c. in the flank, starting from the second week. The parenteral route of administration was preferred for our study in order to avoid first call metabolism by the liver of orally-administered lipids.

The onset of tumours was monitored by daily inspection and by palpation of the mammary regions twice weekly. At week 22 of the experiment, all surviving animals were sacrificed, autopsies were performed and the tumours were dissected and examined histologically.

### In vivo *experiment with iodostearic acid*

A similar investigation was performed using iodostearic acid in place of stearic acid, since it is more readily soluble in lipid solvent than the parent compound. Oleic acid was commercially purchased (Sigma Chemicals, UK) and iodinated by passing dry hydrogen iodide gas in nitrogen through oleic acid at 4°C. Excess iodine was removed at the end of the reaction by the addition of an excess of sodium thiosulphate. The product of these reactions was a mixture of 9 iodo-octadecanoic, 10 iodo-octadecanoic and 9, 10 di-iodo-octadecanoic acids. These can be collectively referred to as iodinated stearic acid.

Fifty-nine female Sprague–Dawley rats weighing ~200 g each were divided into 3 groups. The first group ($n=15$) was injected with NMU alone. The second group ($n=21$) received NMU and alpha$_2$ interferon (Schering). The third group ($n=23$) received NMU plus iodostearic acid dissolved in liquid paraffin. NMU in 3% acetic acid was dissolved in distilled water ($20\,mg\,ml^{-1}$) and given in two i.v. injections of $7\,mg\,100\,g^{-1}$ body wt, the second injection following three weeks after the first. Alpha$_2$ interferon was administered i.m. in a dose of $80{,}000\,IU\,kg^{-1}$ twice each week throughout the experiment. Iodostearic acid was given s.c. in a dose of 0.5 mg twice a week throughout the experiment starting from the 5th week.

Tumour onset was monitored as in the previous experiment. Throughout the experiment only rats that had ulcerated tumours or developed cachexia and marked weakness were sacrificed.

### *Gas-liquid chromatography analysis of rat erythrocytes*

When rats were killed, blood was withdrawn via cardiac puncture and collected in EDTA bottles. Blood was withdrawn from living rats (without tumour) via the tail vein. The aim of this investigation was two-fold. First, to study the possible reduction of stearic to oleic acid ratio in the erythrocytes of rats during chemical carcinogenesis. Second, to investigate whether iodostearic acid inhibited the stearic acid desaturation phenomenon in the tumour-free animals.

Rat erythrocytes were separated by centrifugation. Total lipid extraction was carried out following the method described by MacGee (1974). The extracts were analysed blind using temperature-programmed (160°C to 260°C at 4° per min) gas liquid chromatography of the fatty acid methyl-esters utilising a 2.1 mm x 2 m ID glass column packed with 3% SP-2310/2% SP-2300 on 100/120 mesh chromosorb W (Supelco Inc). Using this method it was possible to separate C16, C18, C20 and C22 fatty acids. The ratio of stearic: oleic fatty acids was taken from the GLC tracing and was expressed as the saturation index (SI). Comparison of indices was made using the Mann–Whitney test.

### Results

### *Clonogenic assays*

Table I demonstrates that stearic acid at a dose of $10\,\mu g\,ml^{-1}$, caused significant inhibition of colony formation in the 4 rat hepatoma cell lines. It failed to inhibit mammary carcinoma (Sp22) or primary fibroblast colony development. Table II shows that stearic acid inhibited colony formation by the human 833 K, RT112 and HFL cell lines, in a dose-related fashion The ID 70 (i.e., dose needed to cause 70% colony formation inhibition) were 2.8, 3.2 and $8.6\,\mu g\,ml^{-1}$ for the 833 K, RT112 and HFL cells lines respectively.

### In vivo *experiment with stearic acid*

Nineteen of the 20 rats in the NMU alone group developed mammary tumours by week 16 of the experiment, with a mean latent period of 72 days. These 19 rats had a total of 51 tumours, giving a mean of 2.68 tumours/rat, range 1–5. The range of tumour weight/rat was 5 g to 47.8 g with a mean of 23.6/rat, excluding the tumour free rats.

Five of the 10 rats in the NMU plus stearic acid group developed mammary tumours by week 16 of the experiment, with a mean latent period of 74 days. These 5 rats had 7 tumours between them, with a mean of 1.4 tumours/rat (compared to NMU alone group $P<0.001$). The range of tumour weight/rat was 4.2 g to 21.2 g, with an average of 16.4 g/rat ($P<0.01$ compared to NMU alone group). By week 22, 19 of the 20 rats in the NMU group were dead with tumours, in contrast with only 2 of 10 rats in the NMU + stearic acid group. Of the remaining 8 that were killed, only 3 had tumours and 5 were tumour-free.

All tumours were examined histologically and were adenocarcinomas.

### In vivo *experiment with iodostearic acid*

Figure 1 shows the results of this experiment. In the NMU alone group one rat died following the first injection of carcinogen. Of the remaining 14 animals, all developed tumours with a mean latent period of 74 days. These rats had 54 tumours between them, giving a mean of 3.8 tumours/rat (range 1–6). Tumour weight/rat ranged from 5 g to 53.5 g with a mean of 23.6 g tumour/rat.

Two rats in the NMU plus interferon group died following carcinogen injection. All the remaining 19 rats developed tumour, with a mean latent period of 77 days. The rats with tumour had 63 tumours between them (range

**Figure I** Percentage of tumour free animals in relation to treatment: NMU; ○ NMU + interferon; NMU + iodostearic acid.

**Table I** Tumour cell colony inhibition test by stearic acid using rat cell lines.

| *Target cells* | *Treatment* | | *Mean no colonies ± SE* | *% plating efficiency* | *% colony formation relative to medium control* | *P value*[a] |
|---|---|---|---|---|---|---|
| Sp22 | medium control | | 61.0 ± 6 | 30.5 | | |
| Rat | 0.5% ethanol | | 61.5 ± 4 | 30.7 | | |
| mammary | stearic acid | 1 ng ml⁻¹ | 54.0 ± 10 | 27.0 | 83 | NS |
| carcinoma | | 10 ng ml⁻¹ | 55.7 ± 3 | 27.8 | 91 | NS |
| | | 100 ng ml⁻¹ | 52.5 ± 6 | 26.2 | 86 | NS |
| | | 1 μg ml⁻¹ | 56.7 ± 3 | 28.3 | 93 | NS |
| | | 10 μg ml⁻¹ | 58.7 ± 1 | 29.3 | 96 | NS |
| | | 50 μg ml⁻¹ | 51.2 ± 6 | 25.6 | 84 | NS |
| Primary | medium control | | 55.0 ± 4.6 | 27.9 | | |
| rat | 0.5% ethanol | | 44.7 ± 1.8 | 22.3 | | |
| fibroblasts | stearic acid | 1 ng ml⁻¹ | 49.5 ± 9.5 | 24.7 | 90.0 | NS |
| (from | | 10 ng ml⁻¹ | 59.7 ± 3.1 | 29.8 | 108.5 | NS |
| alveolar | | 100 ng ml⁻¹ | 47.2 ± 9.4 | 23.6 | 85.8 | NS |
| tissue) | | 1 μg ml⁻¹ | 48.5 ± 9.2 | 24.2 | 88.2 | NS |
| | | 10 μg ml⁻¹ | 52.0 ± 7.7 | 26.0 | 96.5 | NS |
| | | 50 μg ml⁻¹ | 47.5 ± 6.0 | 23.7 | 86.4 | NS |
| D23 | medium control | | 41.8 ± 3.5 | 20.9 | | |
| Rat hepatoma | 0.5% ethanol | | 46.5 ± 1.1 | 23.2 | | |
| | stearic acid | 10 ng ml⁻¹ | 42.3 ± 1.6 | 21.1 | 101.2 | NS |
| | | 100 ng ml⁻¹ | 37.0 ± 1.5 | 17.5 | 88.5 | NS |
| | | 1 μg ml⁻¹ | 28.0 ± 1.5 | 14.0 | 67.0 | <0.02 |
| | | 10 μg ml⁻¹ | 28.0 ± 2.9 | 14.0 | 67.0 | <0.02 |
| | | 50 μg ml⁻¹ | 10.0 ± 2.6 | 5.0 | 23.9 | <0.03 |
| D261 | medium control | | 33.0 ± 5.1 | 16.5 | | |
| Rat hepatoma | 0.5% ethanol | | 30.0 ± 2.4 | 15.0 | | |
| | stearic acid | 1 μg ml⁻¹ | 21.0 ± 3.0 | 10.6 | 64.5 | <0.05 |
| | | 10 μg ml⁻¹ | 18.0 ± 1.5 | 9.0 | 54.5 | <0.01 |
| | | 50 μg ml⁻¹ | 6.0 ± 3.0 | 3.0 | 18.1 | <0.001 |
| D262A | medium control | | 245.0 ± 11.9 | 122.5 | | |
| Rat hepatoma | 0.5% ethanol | | 236.7 ± 8.2 | 118.3 | | |
| | stearic acid | 10 ng ml⁻¹ | 216.0 ± 8.8 | 108.0 | 88.0 | NS |
| | | 100 ng ml⁻¹ | 247.8 ± 8.3 | 123.9 | 101.1 | NS |
| | | 1 μg ml⁻¹ | 203.8 ± 18.1 | 101.9 | 83.2 | NS |
| | | 10 μg ml⁻¹ | 190.3 ± 13.6 | 95.2 | 74.7 | <0.05 |
| | | 50 μg ml⁻¹ | 79.3 ± 7.1 | 39.6 | 32.4 | <0.002 |
| D262B | medium control | | 37.0 ± 4.0 | 18.5 | | |
| Rat hepatoma | 0.5% ethanol | | 34.0 ± 9.5 | 17.0 | | |
| | stearic acid | 10 ng ml⁻¹ | 40.3 ± 1.8 | 20.1 | 108.9 | NS |
| | | 100 ng ml⁻¹ | 41.0 ± 1.8 | 20.5 | 110.8 | NS |
| | | 1 μg ml⁻¹ | 31.0 ± 5.8 | 15.5 | 83.8 | NS |
| | | 10 μg ml⁻¹ | 25.0 ± 2.8 | 12.5 | 67.6 | <0.05 |
| | | 50 μg ml⁻¹ | 18.0 ± 4.5 | 9.0 | 48.6 | <0.02 |

[a]Significance of difference between treated dishes and medium controls (student *t*-test); NS = Not Significant.

**Table II** Tumour cell colony inhibition test by stearic acid using human cell lines.

| *Stearic acid concentration* | *Colony-forming (± standard error)* | | |
|---|---|---|---|
| | *RT112* | *833 K* | *HFL* |
| 1 | 92.0% ± 13.5 | 81.5% ± 3.0 | — |
| 2 | 86.0% ± 10.5 | 70.4% ± 12.0 | 101.3% ± 9.3 |
| 4 | 45.0% ± 6.6 | 27.5% ± 2.0 | 103.1% ± 8.8 |
| 6 | 4.6% ± 0.7 | 0.9% ± 0.5 | 92.8% ± 5.9 |
| 8 | 0.3% ± 0.3 | 0% | 77.3% ± 6.0 |
| 10 | 0% | 0% | 10.5% ± 2.5 |
| 12 | 0% | 0% | 0% |

1–6) with a mean of 3.3 tumours/rat. Tumour weight/rat ranged from 3.8 g to 34.5 g with a mean of 19 g/rat. None of these results were appreciably different from controls.

In the NMU group treated with iodostearic acid, 4 rats died following carcinogen injection. Of the remaining 19 rats, 8 developed tumour with a mean latent period of 76 days. The 8 rats had a mean of 2.7 tumours/rat, with a range of 1–5. Four had extensive tumours (>4 cm diam.), and 2 had tumours between 2–4 cm. The remaining two rats had massive tumours (>4 cm) which regressed subsequently to less than 2 cm on continued treatment with iodostearic acid. At week 22, eleven of the 19 rats treated with NMU plus iodostearic acid were still alive and without tumour.

All the tumours examined histologically were adenocarcinomas.

### *Gas-liquid chromatography analysis of rat erythrocytes*

Figure 2 shows the mean and standard deviation of the SI in each group of rats. The mean SI of the normal saline group was 2.0 ± 0.3. In the NMU group, the erythrocyte SI fell consistently in all rats ($P < 0.001$) to a mean of 1.09 ± 0.28. Similarly, the SI was significantly reduced in rats receiving interferon (mean = 1.1 ± 0.16). By contrast, those rats receiving iodostearic acid that were tumour-free had an SI of 2.12 ± 0.42. Moreover, the tumour-bearing animals receiving iodostearic acid had an SI (mean 1.79 ± 0.33) that was higher than the group with NMU alone ($P < 0.002$), but lower than that of tumour-free animals.

### Discussion

This study has shown that stearic acid significantly inhibits the colony-forming ability of some tumour cell lines, in a

**Figure 2** Changes in saturation index of red blood cell membranes.

dose-related manner. Although human cancer cells appear more sensitive to stearic acid than rat cancer cells, the methodology and culture conditions differed. Other workers have shown a similar effect *in vitro* with a non selective incorporation of fatty acids into cell phospholipids (Doi *et al.*, 1978; Wicha *et al.*, 1979). In those studies the addition of oleic acid had either no effect or stimulated cell division. An important feature of our work has been the demonstration that parenteral administration of stearic acid *in vivo* can prevent the development of tumours in an experimental animal model. We have also provided indirect evidence that inhibition of mammary carcinogenesis is linked to the maintenance of a normal saturation index within the cell membranes. By preserving the ratio of saturated to unsaturated fatty acid, the membrane rigidity remains normal and cell division is inhibited. Those animals that developed tumours despite treatment with stearic acid had significantly fewer and smaller tumours than the animals given carcinogen alone. Their saturation index in erythrocyte membranes was lower than that of tumour-free animals, but was still significantly higher than that of rats given carcinogen without stearic acid. These observations on the erythrocyte saturation index were the same whether the rats were injected with stearic acid or iodostearic acid (data not included).

We have previously shown (Wood *et al.*, 1985) that a decrease in the saturation index of red blood cell membranes is a characteristic finding in patients with a variety of cancers. That this is a reversible change is shown by the return to normal of the saturation index after surgical excision of the tumour, and a subsequent fall with tumour recurrence. Erythrocytes were chosen because they are end-stage non-dividing cells with only one plasma membrane. The extraction procedure used measured the total fatty lipids of the cell membrane rather than the free fatty acids. We have also shown that the drop in saturation index of erythrocytes observed in human cancer can also be seen in rats developing colonic carcinomas following exposure to dimethylhydrazine (Habib *et al.*, 1987*b*), but does not occur in rats with nutritional cachexia (unpublished observation). Similar changes in cell membrane lipid composition also occur in human leucocytes and platelets in cancer patients (Apostolov *et al.*, 1985), suggesting the presence of a desaturation producing factor (DPF) released by the tumour (Habib *et al.*, 1987*a*). We therefore postulate that an alteration in the fatty acid composition of cell membranes may play a role in the carcinogenic process (Habib *et al.*, 1987*b*).

The ability to prevent the alteration in the ratio of fatty acids, as suggested by this study, may either inhibit or merely delay primary oncogenesis. It remains to be shown whether stearic or iodostearic acid have a direct effect on the erythrocyte cell membranes or whether the response is secondary to changes in the bone marrow. It is interesting to note that in two rats tumours actually regressed with continued stearic acid therapy. If confirmed, this finding raises the possibility that in addition to inhibiting cell division, altering the fatty acid structure of the membrane may have a direct anti-tumour effect. Future work will investigate the role of stearic acid and its derivatives in the treatment of neoplasia by studying the effect on established tumours growing as transplants.

Our thanks to Miss K. Bradley for typing the manuscript.

### References

APOSTOLOV, K., BARKER, W., CATOVSKY, D., GOLDMAN, J. & MATUTES, E. (1985). Reduction in the stearic to oleic acid ratio in leukaemic cells – A possible chemical marker of malignancy. *Blut.*, **50**, 349.

APOSTOLOV, K., BARKER, W., WOOD, C.B., HABIB, N.A., JEFFRIES, D. & FORSTER, S.M. (1987). Fatty acid saturation index in peripheral blood cell membrane of AIDS patients. *Lancet*, **i**, 695.

APOSTOLOV, K. & BARKER, W. (1981). The effects of interferon on the fatty acids in uninfected cells. *FEBS Lett.*, **126**, 261.

CHAN, P.-C., HEAD, J.F., COHEN, L.A. & WYNDER, E.L. (1977). Influences of dietary fat on the induction of mammary tumours by *N*-nitroso-methylurea: Associated hormone changes and differences between Sprague–Dawley and F344 rats. *J. Natl Cancer Inst.*, **59**, 1279.

COOPER, R.A. (1977). Abnormalities of cell membrane fluidity in the pathogenesis of disease. *N. Engl. J. Med.*, **297**, 371.

CORVIN, L.M., HUMPHREY, L.P. & SHLOSS, J. (1977). Effect of lipid on the expression of cell transformation. *Exp. Cell Res.*, **108**, 341.

DOI, O., DOI, F., SCHROEDER, F., ALBERTS, S.W. & VAGELOS, P.R. (1978). Manipulation of fatty acid composition of membrane phospholipid and its effect on cell growth in mouse LM cells. *Biochem. Biophys. Acta.*, **509**, 239.

GULLINO, P.M., PETTIGREW, H.M. & GRANTHAM, E.H. (1975). *N*-nitroso-methylurea as mammary gland carcinogen in rats. *J. Natl Cancer Inst.*, **54**, 401.

HABIB, N.A., HERSHMAN, M.J., APOSTOLOV, K., BARKER, W. & WOOD, C.B. (1987). Desaturation of cell membrane fatty acids by urine from patients with cancer. *Surg. Res. Comm.*, **1**, 111.

HABIB, N.A., HERSHMAN, M.J., SALEM, R., APOSTOLOV, K. & WOOD, C.B. (1987). Increased erythrocyte stearic acid desaturation in rats with chemically induced colorectal carcinomas. *Int. J. Colorectal Dis.*, **2**, 12.

MACGEE, J. (1974). Preparation of methyl esters from the saponifiable fatty acids in small biological specimens for GLC analysis. *J. Chromatog.*, **100**, 35.

MOUNTFORD, C.E., MAY, L.G., WILLIAMS, G.P. & 7 others (1986). Classification of human tumours by high resolution magnetic resonance spectroscopy. *Lancet*, **i**, 651.

PETITOU, M., TUY, F., ROSENFLED, C. & 5 others (1978). Decreased microviscosity of membrane lipids in leukaemic cells: Two possible mechanisms. *Proc. Nat. Acad. Sci. USA.*, **75**, 2306.

PREUSSMANN, R. & STEWART, B.W. (1984). *N*-Nitroso carcinogens. *Am. Chem. Soc. Mono.*, **182**, 643.

SANDEMANN, H., JR. (1979). Regulation of membrane enzymes by lipids. *Biochem. Biophys. Acta.*, **515**, 209.

WICHA, M.S., LIOTTA, L.A. & KIDWELL, W.R. (1979). Effects of free fatty acids on the growth of normal and neoplastic rat mammary epithelial cells. *Cancer Res.*, **39**, 426.

WORMAN, C.P., BARKER, W.R. & APOSTOLOV, K. (1987). Saturation index of blood cell membrane fatty acids before and after IFN treatment in hairy-cell leukaemia (HCL). *Leukaemia*, **1**, 379.

WOOD, C.B., HABIB, N.A., APOSTOLOV, K. & 4 others (1985). Reduction in the stearic to oleic acid ratio in human malignant liver neoplasms. *Eur. J Surg. Oncol.*, **11**, 347.

WOOD, C.B., HABIB, N.A. & THOMPSON, A. (1985). Increase of oleic acid in erythrocytes associated with malignancies. *Br. Med. J.*, **291**, 163.